# Self-templated Synthesis of Nickel Silicate Hydroxide/Reduced Graphene Oxide Composite Hollow Microspheres as Highly Stable Supercapacitor Electrode Material

**DOI:** 10.1186/s11671-017-2094-9

**Published:** 2017-05-04

**Authors:** Yanhua Zhang, Wenjie Zhou, Hong Yu, Tong Feng, Yong Pu, Hongdong Liu, Wei Xiao, Liangliang Tian

**Affiliations:** Research Institute for New Materials Technology, Chongqing University of Arts and Sciences, Yongchuan, Chongqing, 402160 China

**Keywords:** Nickel silicate hydroxide, Graphene, Hollow structure, Supercapacitor, Self-template, Hydrothermal synthesis

## Abstract

Nickel silicate hydroxide/reduced graphene oxide (Ni_3_Si_2_O_5_(OH)_4_/RGO) composite hollow microspheres were one-pot hydrothermally synthesized by employing graphene oxide (GO)-wrapped SiO_2_ microspheres as the template and silicon source, which were prepared through sonication-assisted interfacial self-assembly of tiny GO sheets on positively charged SiO_2_ substrate microspheres. The composition, morphology, structure, and phase of Ni_3_Si_2_O_5_(OH)_4_/RGO microspheres as well as their electrochemical properties were carefully studied. It was found that Ni_3_Si_2_O_5_(OH)_4_/RGO microspheres featured distinct hierarchical porous morphology with hollow architecture and a large specific surface area as high as 67.6 m^2^ g^–1^. When utilized as a supercapacitor electrode material, Ni_3_Si_2_O_5_(OH)_4_/RGO hollow microspheres released a maximum specific capacitance of 178.9 F g^−1^ at the current density of 1 A g^−1^, which was much higher than that of the contrastive bare Ni_3_Si_2_O_5_(OH)_4_ hollow microspheres and bare RGO material developed in this work, displaying enhanced supercapacitive behavior. Impressively, the Ni_3_Si_2_O_5_(OH)_4_/RGO microsphere electrode exhibited outstanding rate capability and long-term cycling stability and durability with 97.6% retention of the initial capacitance after continuous charging/discharging for up to 5000 cycles at the current density of 6 A g^−1^, which is superior or comparable to that of most of other reported nickel-based electrode materials, hence showing promising application potential in the energy storage area.

**Electronic supplementary material:**

The online version of this article (doi:10.1186/s11671-017-2094-9) contains supplementary material, which is available to authorized users.

## Background

To ease the energy crisis and environmental problems, there is an important and urgent need to develop clean and sustainable power sources as well as advanced energy conversion and storage devices [1]. Supercapacitors, usually known as electrochemical capacitors, have attracted tremendous attention owing to their higher energy density than traditional dielectric capacitors, higher power density than batteries, rapid charge/discharge rate, and quite long cycle life [2]. The exploration of high-performance electrode materials is a crucial challenge for the construction and application of supercapacitors. Up to now, a large number of supercapacitor electrode materials with different components, morphologies, and architecture such as nanostructured carbonaceous matter (e.g., porous carbon, graphene network, carbon nanotubes), metal sulfides (e.g., MoS_2_, Ni_3_S_2_, WS_2_), metal oxides (e.g., MnO_2_, RuO_2_, CeO_2_), metal hydroxides (e.g., Co(OH)_2_, Ni(OH)_2_), conducting polymers (e.g., polyaniline, polypyrrole), and their hybrid composites have been well fabricated [2–8]. Unfortunately, most of them suffer from one or more problems like high cost, complicated preparative process, limited specific capacitance, unsatisfactory cycling stability, and low rate capability. Among these disadvantages, the inferior cycling stability is particularly acute, which severely restricts their further practical applications in the supercapacitor field [9]. Consequently, it remains a challenging task to develop highly stable electrode materials with excellent supercapacitive behavior through facile and cost-effective strategies.

As a typical member of metal silicate hydroxides, nickel silicate hydroxide (Ni_3_Si_2_O_5_(OH)_4_) has a layered structure formed by outer octahedral Ni(II)O_6_ sheets and inner tetrahedral SiO_4_ sheets [10]. Thanks to the earth abundance and environmental friendliness, Ni_3_Si_2_O_5_(OH)_4_ has been widely utilized as adsorbents for heavy metal ions and organic dyes, carriers for drug release, molecular sieves, and catalyst supports [10–14]. However, its application as electroactive materials is quite limited because of its intrinsic poor electronic conductivity [10]. Despite this drawback, the layered structure of Ni_3_Si_2_O_5_(OH)_4_ still endows it with an appealing feature for electrochemical applications, since such structure could provide numerous well-defined multichannels for fast mass transfer, which is a critical factor during electrochemical reactions [10]. To improve the conductivity of Ni_3_Si_2_O_5_(OH)_4_-based materials, hybridization of Ni_3_Si_2_O_5_(OH)_4_ with a conductive matrix including reduced graphene oxide and carbon nanotubes has been recently achieved, and the resulting composites were successfully used in electrocatalytic water oxidation and lithium-ion batteries [10, 15–17]. Nevertheless, the report with respect to the application of Ni_3_Si_2_O_5_(OH)_4_-based materials in supercapacitor remains rare.

**Fig. 1:**
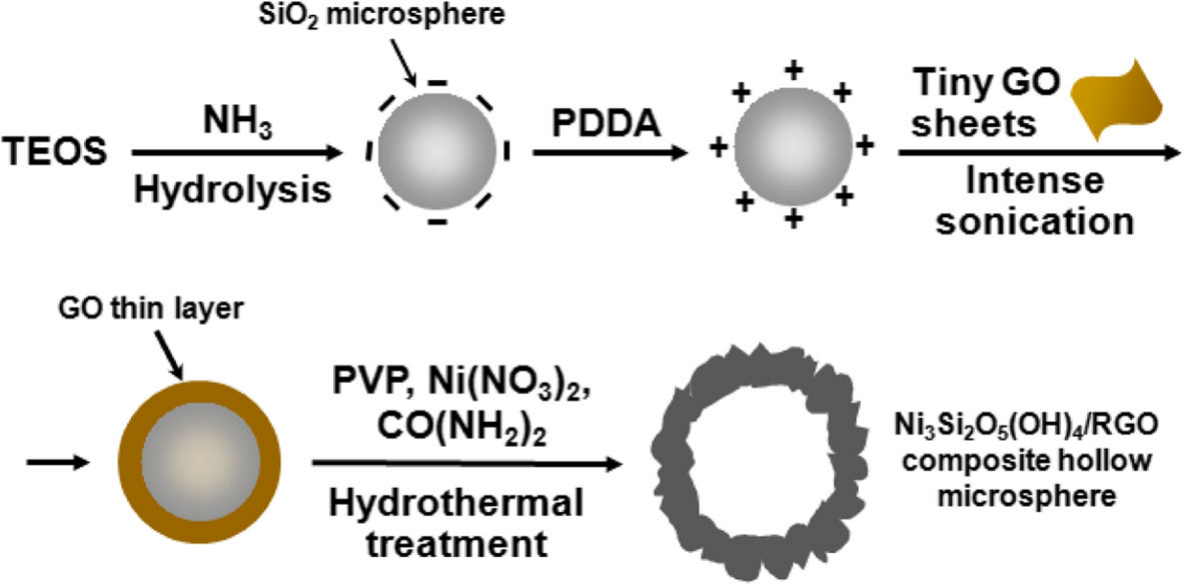
Schematic representation of the fabrication of hierarchical flower-like Ni_3_Si_2_O_5_(OH)_4_/RGO composite hollow microspheres

Graphene, a single layer of graphite, has been regarded as one of the most promising materials due to its attractive physicochemical properties and functions like light weight, exceptional electronic conductivity, and splendid chemical stability [17]. Accordingly, integration of graphene or reduced graphene oxide (RGO) with other inorganic species to boost electrochemical behavior has become an effective strategy, and a variety of graphene- or RGO-containing hybrids (e.g., hollow-structured MoS_2_/RGO microspheres, RGO-wrapped polyaniline nanowires, nanocubic Co_3_O_4_/RGO composites) with reinforced supercapacitive performance have been explored as well [4, 18, 19]. Over the past few years, self-assembly of graphene oxide (GO) sheets on solid substrates via electrostatic interaction has been demonstrated to be a versatile way to prepare GO- and RGO-based composites [20]. By means of this methodology, we have pioneered the fabrication of highly water-dispersible GO-encapsulated SiO_2_ microspheres (Fig. 1). The excellent aqueous dispersity of the resultant SiO_2_/GO composite microspheres enabled them to be readily modified or treated for further functionalization [4, 21, 22]. Herein, we take advantage of this point and utilize them as the template and silicon source to prepare flower-like nickel silicate hydroxide/reduced graphene oxide (Ni_3_Si_2_O_5_(OH)_4_/RGO) composite hollow microspheres with a hierarchical porous structure in one pot. As illustrated in Fig. 1, SiO_2_/GO microspheres underwent a hydrothermal process in the presence of polyvinylpyrrolidone, nickel nitrate, and urea, during which the SiO_2_ inner core reacted with nickel cations to produce Ni_3_Si_2_O_5_(OH)_4_ in alkaline condition and its deposition, growth, and crystallization on substrate microspheres together with the reduction of GO to RGO were synchronously accomplished, giving rise to the final product of Ni_3_Si_2_O_5_(OH)_4_/RGO composite hollow microspheres. When employed as a supercapacitor electrode material, the synthesized Ni_3_Si_2_O_5_(OH)_4_/RGO microspheres released a maximum specific capacitance of 178.9 F g^−1^ at the current density of 1 A g^−1^ in a three-electrode system and maintained 97.6% of the initial capacitance after repetitive charging/discharging at the current of 6 A g^−1^ over 5000 cycles, exhibiting outstanding long-term cycling stability and durability.

## Methods

### Materials and Reagents

Ammonium hydroxide (25 wt%), tetraethyl orthosilicate, poly(diallyldimethylammonium) chloride (PDDA), nickel nitrate hexahydrate, urea, polyvinylpyrrolidone (PVP) with an average molecular weight of 40,000, acetylene black, *N*-methyl-2-pyrrolidone (NMP), nickel foam, potassium hydroxide, and polyvinylidene fluoride (PVDF) were purchased from J&K Co., Ltd. (Shanghai, China). Commercial tiny GO sheets with the lateral size less than 200 nm were provided by Nanjing JCNANO Technology Co., Ltd. (Nanjing, China). All other chemicals were guaranteed reagents and directly used as received.

### Synthesis of Ni_3_Si_2_O_5_(OH)_4_/RGO Composite Hollow Microspheres

Monodisperse colloidal SiO_2_ microspheres with the diameter of ~300 nm were first prepared based on a modified Stöber method (see Additional file 1 for experimental details). Subsequently, GO-encapsulated SiO_2_ microspheres (i.e., SiO_2_/GO composite microspheres) were fabricated by sonication-assisted interfacial self-assembly of tiny GO sheets on cationic polyelectrolyte-decorated SiO_2_ microspheres (i.e., PDDA-modified SiO_2_ microspheres) through electrostatic interaction (see Additional file 1 for experimental details). Ni_3_Si_2_O_5_(OH)_4_/RGO hollow microspheres were one-step hydrothermally synthesized through a self-template route. Typically, 20 mg of SiO_2_/GO microspheres were dispersed in 12 mL of water, followed by introduction of 8 mL of mixed aqueous solution containing 80 mg of nickel nitrate hexahydrate, 0.6 g of PVP, and 1 g of urea under sonication. The resulting reaction mixture was then poured into a stainless autoclave (50 mL of capacity) and sealed, which was subsequently allowed to undergo a hydrothermal reaction at 180 °C for 12 h. During the hydrothermal process, SiO_2_ reacted with nickel ions and urea to yield Ni_3_Si_2_O_5_(OH)_4_, which was grown on the substrate microspheres. At the same time, the GO component was hydrothermally reduced to RGO, hence resulting in the generation of flower-like Ni_3_Si_2_O_5_(OH)_4_/RGO composite hollow microspheres in one pot. After that, the product was separated and washed with abundant water, followed by drying and annealing at 600 °C for 2 h in Ar atmosphere. To demonstrate the role of PVP in the hydrothermal synthetic system, a Ni_3_Si_2_O_5_(OH)_4_/RGO hybrid material was similarly synthesized according to the above procedure but without the introduction of PVP. As control, contrastive bare Ni_3_Si_2_O_5_(OH)_4_ hollow microspheres were hydrothermally fabricated by using pure SiO_2_ microspheres as the template, followed by the same annealing treatment. In addition, bare RGO material was also prepared through hydrothermal reduction of tiny GO sheets at 180 °C for 12 h.

### Characterizations

Powder X-ray diffraction (XRD) patterns with the scanning range from 10° to 70° were obtained on a Bruker D8 ADVANCE diffractometer. Field emission scanning electron microscopy (FESEM) inspection was performed on a Hitachi SU8010 microscope working at the acceleration voltage of 3 kV. Transmission electron microscopy (TEM) observation was carried out on a JEOL JEM-2100F microscope operating at the acceleration voltage of 200 kV and equipped with an energy-dispersive spectroscopy (EDS) system. X-ray photoelectron spectra (XPS) were recorded on a VG ESCALAB MARK II instrument. Raman spectra were collected from a HORIBA Scientific Raman spectrometer with the excitation source of 532-nm laser line. Nitrogen adsorption–desorption isotherms were recorded on a Micromeritics ASAP 2020 apparatus at −196 °C, and the specific surface area of the samples was calculated by the Brunauer–Emmett–Teller (BET) model.

### Electrochemical Measurements

All the electrochemical tests were done on a CHI 760E electrochemical workstation (CH Instruments, Inc., Shanghai, China) with a three-electrode system by employing aqueous solution of 2 M KOH as the electrolyte. Hg/HgO electrode, platinum foil, and nickel foam substrate coated with active material were used as the reference electrode, counter electrode, and working electrode, respectively. To fabricate the working electrode, the active material was mixed with acetylene black and PVDF at the weight ratio of 80:10:10. Then, NMP was added into the mixture, followed by gentle grinding to generate a homogeneous slurry. After that, the resulting slurry was pasted onto a nickel foam current collector with the area of 1 cm × 1 cm, followed by drying at 60 °C overnight in a vacuum oven, and the loading amount of active material on the working electrode was ~2.5 mg. Cyclic voltammetry (CV) curves were recorded in the potential window between 0.15 and 0.65 V at various scanning rates. Galvanostatic charge/discharge (GCD) measurements were done in the potential range from 0.2 to 0.6 V at a series of current densities. Electrochemical impedance spectroscopy (EIS) was carried out in the frequency range from 0.01 to 100,000 Hz at open-circuit potential with an ac perturbation of 5 mV.

## Results and Discussion

### Material Characterizations

**Fig. 2:**
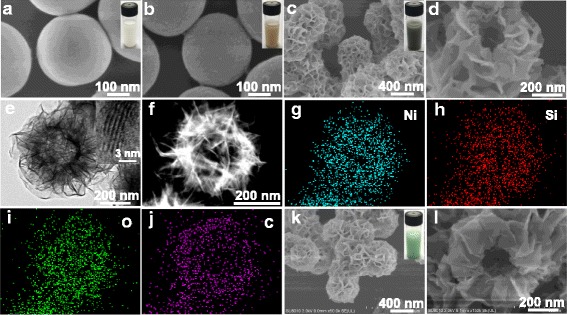
**a**, **b** FESEM images of pristine SiO_2_ and SiO_2_/GO microspheres, showing the smooth and relatively rougher external surface, respectively; the *insets* in **a** and **b** are digital photographs of the corresponding aqueous suspensions of SiO_2_ and SiO_2_/GO microspheres, which are pure *white* and *yellow brown* in color, respectively. **c**, **d** FESEM images of as-prepared Ni_3_Si_2_O_5_(OH)_4_/RGO composite hollow microspheres at low and high magnifications, respectively; the *inset* in **c** is a digital photograph of their aqueous dispersion, showing dark black in color. **e** TEM image of an individual Ni_3_Si_2_O_5_(OH)_4_/RGO hollow microsphere; the *inset* is an HRTEM image of a random nanoflake located on its surface, displaying the (002) lattice plane. **f** STEM image of a single Ni_3_Si_2_O_5_(OH)_4_/RGO hollow microsphere. **g**–**j** EDS mapping images of elements Ni, Si, O, and C for the Ni_3_Si_2_O_5_(OH)_4_/RGO microsphere shown in **f. k**, **l** FESEM images of bare Ni_3_Si_2_O_5_(OH)_4_ hollow microspheres at low and high magnifications, respectively; the *inset* in **k** is a digital photograph of their aqueous dispersion, exhibiting bright* green* in color

Figure 2a and Additional file 1: Figure S1a, b are the FESEM images of pure monodisperse SiO_2_ microspheres with the diameter of ~300 nm and a perfect smooth surface, displaying white color (inset of Fig. 2a). Figure 2b and Additional file 1: Figure S1c, d present the FESEM images of SiO_2_/GO microspheres, whose size seems to be unchanged as compared with SiO_2_ microspheres while whose apparent color becomes yellow brown (inset of Fig. 2b). Also, the outer surface of SiO_2_/GO microspheres seems to be slightly rougher and some twisted crumples can be identified (Fig 2b and Additional file 1: Figure S1d), which should arise from the encapsulation of tiny GO sheets on substrate microspheres. These results confirm the successful sonication-assisted interfacial self-assembly of tiny GO sheets on the positively charged SiO_2_ microspheres by virtue of electrostatic interaction. Ni_3_Si_2_O_5_(OH)_4_/RGO composite hollow microspheres were one-pot prepared by hydrothermal treatment of the SiO_2_/GO microsphere template in the presence of nickel nitrate, urea, and PVP, and their morphology was carefully inspected. Compared with the pristine SiO_2_ and SiO_2_/GO composite microspheres, Ni_3_Si_2_O_5_(OH)_4_/RGO microspheres are bigger in size (~600 nm in diameter), and their external surface is composed of plenty of highly curved and wrinkled nanoflakes with the thickness of tens of nanometers (Fig. 2c, d), which should originate from the homogeneous deposition, coverage, and growth of Ni_3_Si_2_O_5_(OH)_4_ on the substrate microspheres, leading to the hierarchical porous architecture with a flower-like shape. Meanwhile, differing from the apparent colors of pristine SiO_2_ and SiO_2_/GO composite microspheres, Ni_3_Si_2_O_5_(OH)_4_/RGO microspheres show a dark color (inset of Fig. 2c), and such deep color is owing to the presence of the RGO component within the sample. Figure 2d exhibits the FESEM image of a typical Ni_3_Si_2_O_5_(OH)_4_/RGO microsphere with a broken shell, revealing the hollow structure, which was further verified by the following TEM and scanning TEM (STEM) examinations. As exhibited in Fig. 2e, f, an evident interior cavity with a uniform shell thickness of ~150 nm was found in each well-defined Ni_3_Si_2_O_5_(OH)_4_/RGO microsphere, which is indicative of total removal of the SiO_2_ template but without collapse of the hollow structure. The inset of Fig. 2e is a high-resolution TEM (HRTEM) image of an arbitrary Ni_3_Si_2_O_5_(OH)_4_ nanoflake anchored on a Ni_3_Si_2_O_5_(OH)_4_/RGO microsphere, where the lattice fringes are visible and the interplanar spacing is calculated to be 0.74 nm, agreeing well with the (002) crystal plane of Ni_3_Si_2_O_5_(OH)_4_ [9, 16, 17]. The elemental distribution of the Ni_3_Si_2_O_5_(OH)_4_/RGO microsphere presented in Fig. 2f was further analyzed by the corresponding EDS mappings. As can be clearly seen in Fig. 2g–j, all the Ni, Si, O, and C signals were detected and filled the microsphere area, demonstrating their homogeneous distribution in this sample. Notably, PVP plays an important role in the hydrothermal synthetic system. In the absence of PVP, although a Ni_3_Si_2_O_5_(OH)_4_/RGO hybrid material can be fabricated as well, such composite agglomerated seriously and its spherical morphology was rather inferior to that of Ni_3_Si_2_O_5_(OH)_4_/RGO composite hollow microspheres (Additional file 1: Figure S2). It is assumed that PVP favored the dispersion of substrate microspheres (i.e., the SiO_2_/GO microspheres) and effectively alleviated the agglomeration of products during the hydrothermal process, leading to well-defined Ni_3_Si_2_O_5_(OH)_4_/RGO composite hollow microspheres. Moreover, as a comparison, bare Ni_3_Si_2_O_5_(OH)_4_ hollow microspheres were hydrothermally fabricated by employing pure SiO_2_ microspheres as the template, and the synthetic conditions were identical to those for preparation of Ni_3_Si_2_O_5_(OH)_4_/RGO microspheres. Obviously, their sphere-like shape, hierarchical morphology, and hollow structure are similar to those of the counterpart (Fig. 2k, l), whereas their apparent color shows bright green (inset of Fig. 2k).

**Fig. 3:**
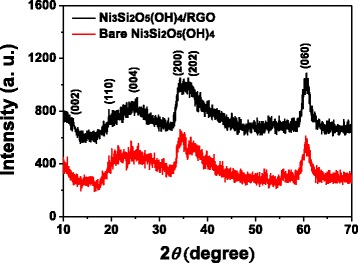
XRD patterns of Ni_3_Si_2_O_5_(OH)_4_/RGO composite hollow microspheres (*black curve*) and bare Ni_3_Si_2_O_5_(OH)_4_ hollow microspheres (*red curve*)

Powder XRD technique was made to characterize the structure and phase information of the products. As shown in the XRD patterns of both Ni_3_Si_2_O_5_(OH)_4_/RGO microspheres and bare Ni_3_Si_2_O_5_(OH)_4_ microspheres (Fig. 3), six diffraction peaks are available at around 2*θ* = 12.0°, 19.6°, 24.5°, 34.2°, 36.7°, and 60.5°, which are well indexed to the (002), (110), (004), (200), (202), and (060) crystal planes of pecoraite Ni_3_Si_2_O_5_(OH)_4_, respectively. These values are in accordance with previous reports and the standard XRD pattern (JCPDS no. 49-1859) as well [9, 10, 15, 17]. Commonly, RGO features a broad diffraction peak at 2*θ* = 25°; nevertheless, in the XRD pattern of Ni_3_Si_2_O_5_(OH)_4_/RGO microspheres, it is likely to be overlapped by the peaks corresponding to the (110) and (004) crystal planes of the Ni_3_Si_2_O_5_(OH)_4_ component as a result of the low content and weak diffraction intensity of RGO within Ni_3_Si_2_O_5_(OH)_4_/RGO microspheres [15, 17].

**Fig. 4:**
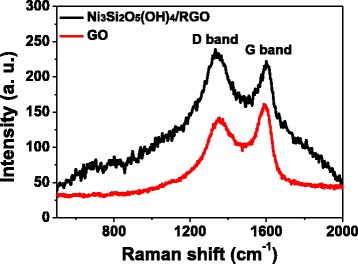
Raman spectra of Ni_3_Si_2_O_5_(OH)_4_/RGO hollow microspheres (*black curve*) and GO sheets (*red curve*), showing the D and G bands

To verify the existence of the RGO component incorporated in Ni_3_Si_2_O_5_(OH)_4_/RGO microspheres, the Raman spectra of Ni_3_Si_2_O_5_(OH)_4_/RGO microspheres and tiny GO sheets were characterized and depicted in Fig. 4. Obviously, there are a couple of bands at around 1350 and 1590 cm^–1^ in both curves, which are ascribed to the characteristic D and G bands of graphene-based species [14, 15, 17]. Generally, the D band arises from the structural defects and edges that damage the symmetry, while the G band refers to the first-order scattering of *E*_*2g*_ phonons [4, 14]. Especially, the peak intensity ratio of the D to the G band (*I*_D_/*I*_G_) is a useful measure to evaluate the graphitization degree of carbon matter [4, 14]. The *I*_D_/*I*_G_ value for Ni_3_Si_2_O_5_(OH)_4_/RGO microspheres is 1.08, which is higher than that for GO sheets (0.88), implying that the reduction of GO to RGO indeed occurred during the hydrothermal process, which was undoubtedly incorporated in the final product of Ni_3_Si_2_O_5_(OH)_4_/RGO microspheres [4, 14, 17].

**Fig. 5:**
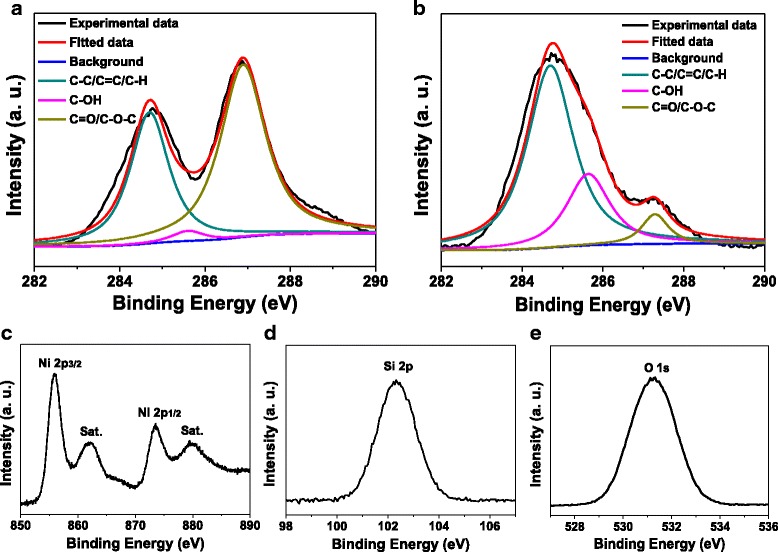
**a** High-resolution XPS of C 1s of tiny GO sheets. **b**–**e** High-resolution XPS of Ni_3_Si_2_O_5_(OH)_4_/RGO hollow microspheres, showing the C 1s, Ni 2p, Si 2p, and O 1s regions, respectively

X-ray photoelectron spectroscopy provides an effective tool for disclosing the surface composition and state of hybrid materials. Figure 5a gives the high-resolution XPS of C 1s of GO sheets. Figure 5b–e shows a set of high-resolution XPS of Ni_3_Si_2_O_5_(OH)_4_/RGO microspheres for the C 1s, Ni 2p, Si 2p, and O 1s regions, respectively. As envisioned, the detected signals suggest the presence of the four elements in the sample. Both of the C 1s spectra can be resolved into three Gaussian fitted peaks. The peak located at 284.6 eV is attributed to the oxygen-free C=C and C–C bonding, whereas the other two peaks found around 285.6 and 287.1 eV are related to diverse oxygen-containing groups including C–OH, O=C, and C−O−C [23, 24]. The relative intensity of oxygen-containing groups in the C 1s spectrum of Ni_3_Si_2_O_5_(OH)_4_/RGO microspheres significantly decreased as compared with that in the C 1s spectrum of tiny GO sheets, once again indicating that the immobilized GO sheets wrapping on the substrate microspheres underwent a drastic loss of oxygen-containing groups during the hydrothermal reaction, leading to its reduction to the RGO component [4, 22, 23]. Figure 5c is the high-resolution XPS of Ni 2p of Ni_3_Si_2_O_5_(OH)_4_/RGO microspheres, where a pair of predominant peaks appear at 856.0 and 873.5 eV, corresponding to the binding energy (BE) of Ni 2p3/2 and Ni 2p1/2, respectively [10, 25]. Two shakeup satellite peaks (denoted as “Sat.” in Fig. 5c) close to the spin-orbit doublets are also visible at 862.0 and 880.1 eV with the BE separation of 18.1 eV [10, 25]. All these data agree well with the reported ones and demonstrate the presence of Ni(II) in this sample [10, 25]. Besides, the high-resolution XPS of Si 2p and O 1s reveal strong peaks at 102.3 and 531.3 eV, respectively, which are typical BE values for metal silicate hydroxides as well and mainly derive from the Ni−Si and Si−O bonding [9, 10].

**Fig. 6:**
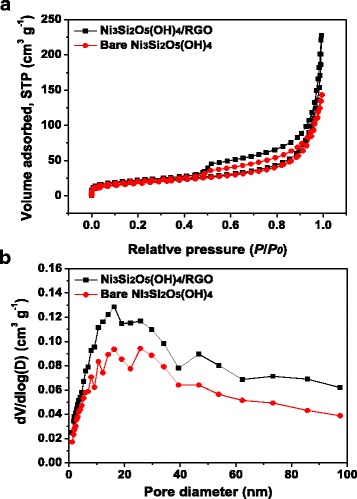
**a** Nitrogen adsorption–desorption isotherms and **b** pore size distribution profiles of Ni_3_Si_2_O_5_(OH)_4_/RGO hollow microspheres (*black curves*) and bare Ni_3_Si_2_O_5_(OH)_4_ hollow microspheres (*red curves*)

The porous feature of Ni_3_Si_2_O_5_(OH)_4_/RGO and bare Ni_3_Si_2_O_5_(OH)_4_ microspheres was surveyed by BET measurements. As shown in their nitrogen adsorption–desorption isotherms (Fig. 6a), both of them can be classified into type IV isotherms with a typical hysteresis loop ranging from 0.5 to 0.9 *P*/*P*_0_ in each of them, suggesting the presence of mesopores in the two specimens [4, 26]. Based on the isotherms, the pore size distribution and specific surface area are deduced according to the Barrett–Joyner–Halenda model, and the corresponding plots are presented in Fig. 6b, which once again manifest the existence of well-developed porosity with an average pore size centered around 20 nm and a wide distribution from micropores to macropores in both samples [2, 26]. Such result is consistent with their FESEM and TEM observations as well (Fig. 2), and the pores are possibly formed by the complex intertwining and stacking among the nanoflakes [27]. Thanks to the hierarchical porous architecture, the specific surface area of Ni_3_Si_2_O_5_(OH)_4_/RGO and bare Ni_3_Si_2_O_5_(OH)_4_ microspheres is as high as 67.6 and 61.6 m^2^ g^–1^, respectively. It is assumed that the larger specific surface area of Ni_3_Si_2_O_5_(OH)_4_/RGO microspheres would increase the contact area between the electrolyte and electrode material, facilitate the mass transport of charged ions, and provide more reactive sites during electrochemical reactions and thus bring about preferable supercapacitive performance [4, 28, 29].

### Electrochemical Investigation

**Fig. 7:**
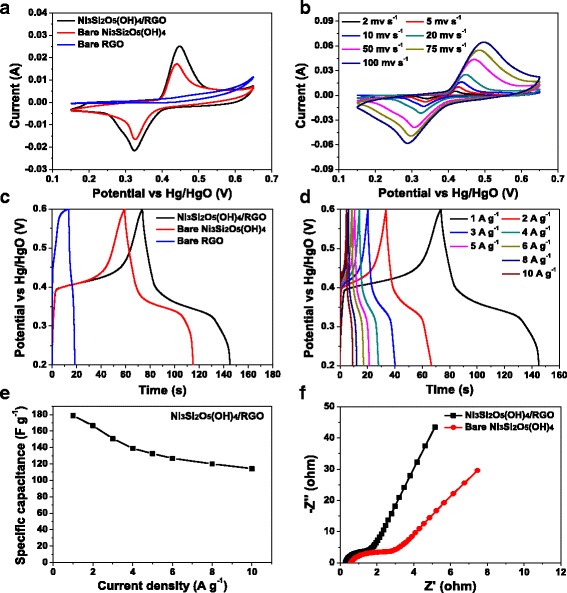
**a** CV curves of Ni_3_Si_2_O_5_(OH)_4_/RGO hollow microspheres (*black curve*), bare Ni_3_Si_2_O_5_(OH)_4_ hollow microspheres (*red curve*), and bare RGO material (*blue curve*) tested at the scanning rate of 20 mV s^–1^ in 2 M KOH. **b** CV curves of Ni_3_Si_2_O_5_(OH)_4_/RGO hollow microspheres tested at a series of different sweeping rates. **c** GCD curves of Ni_3_Si_2_O_5_(OH)_4_/RGO hollow microspheres (*black curve*), bare Ni_3_Si_2_O_5_(OH)_4_/RGO microspheres (*red curve*) and bare RGO material (*blue curve*) at the current density of 1 A g^–1^. **d** GCD curves of Ni_3_Si_2_O_5_(OH)_4_/RGO hollow microspheres measured at a set of varied current densities. **e** Specific capacitance of Ni_3_Si_2_O_5_(OH)_4_/RGO hollow microsphere electrode deduced from the GCD curves depicted in **d** as a function of current density. **f** Nyquist plots of Ni_3_Si_2_O_5_(OH)_4_/RGO hollow microsphere (*black curve*) and bare Ni_3_Si_2_O_5_(OH)_4_ hollow microsphere (*red curve*) electrodes

The electrochemical properties of Ni_3_Si_2_O_5_(OH)_4_/RGO hollow microspheres, bare Ni_3_Si_2_O_5_(OH)_4_ hollow microspheres, and bare RGO material were evaluated by CV and GCD measurements in a three-electrode system employing aqueous solution of 2 M KOH as the electrolyte. Figure 7a displays their CV curves at the sweeping rate of 20 mV s^–1^. There are a pair of redox peaks in both CV curves of Ni_3_Si_2_O_5_(OH)_4_/RGO and bare Ni_3_Si_2_O_5_(OH)_4_ microspheres, which arise from the transition of nickel ions between different oxidation states [10, 27, 30]:

$$ {\mathrm{Ni}}_3^{\mathrm{II}}{\mathrm{Si}}_2{\mathrm{O}}_5{\left(\mathrm{OH}\right)}_4+2{\mathrm{O}\mathrm{H}}^{{\textstyle \hbox{-} }}\leftrightarrow {\mathrm{Ni}}_3^{\mathrm{II}\mathrm{I}}{\mathrm{Si}}_2{\mathrm{O}}_5{\left(\mathrm{OH}\right)}_6+2{\mathrm{e}}^{{\textstyle \hbox{-} }} $$

implying their pseudocapacitive characteristics [27, 30], while there are no obvious redox peaks in the CV curve of bare RGO material, suggesting its electric double-layer capacitance nature [2, 4]. The peak current density and the integral area enclosed by the CV curve of Ni_3_Si_2_O_5_(OH)_4_/RGO hollow microspheres are higher than those of bare Ni_3_Si_2_O_5_(OH)_4_ hollow microspheres and bare RGO material, indicating the best capacitance [4, 9]. Figure 7b is the CV curves of Ni_3_Si_2_O_5_(OH)_4_/RGO hollow microspheres at varied scanning rates of 2–100 mV s^−1^. With elevating sweeping rates, the shape of the CV curves is not remarkably altered and the intensity of redox peaks gradually goes up with only a slight shift toward higher potential, demonstrating that fast electrochemical reactions take place at the interface between the electrolyte and active material and the Ni_3_Si_2_O_5_(OH)_4_/RGO hollow microsphere electrode possesses excellent rate capability [19, 31]. Figure 7c depicts the GCD curves of Ni_3_Si_2_O_5_(OH)_4_/RGO, bare Ni_3_Si_2_O_5_(OH)_4_, and bare RGO electrodes in the potential range of 0.2–0.6 V tested at the current density of 1 A g^−1^. It is not hard to see that the discharge time of the Ni_3_Si_2_O_5_(OH)_4_/RGO microsphere electrode is the longest. Such result is consistent with the CV measurements displayed in Fig. 7a and further confirms its superior supercapacitive behavior. The specific capacitance of a single electrode is able to be obtained on the basis of the equation described as follows:

$$ C= i\cdot \varDelta t/\varDelta V\cdot m $$

where *C* (F g^−1^) stands for the specific capacitance, *i* (A) represents the constant current, *t* (s) is the discharge time, Δ*V* (V) is the potential window, and *m* (g) is the mass of active material [4, 9, 27]. Therefore, the *C* of Ni_3_Si_2_O_5_(OH)_4_/RGO microspheres at the current density 1 A g^−1^ is deduced to be 178.9 F g^−1^, which is clearly higher than that of bare Ni_3_Si_2_O_5_(OH)_4_ microspheres (138.4 F g^−1^) and bare RGO material (12.2 F g^−1^). Figure 7d gives its GCD curves at a group of different current densities, based on which the *C* of the Ni_3_Si_2_O_5_(OH)_4_/RGO microsphere electrode is calculated to be 178.9, 166.5, 150.8, 138.9, 132.5, 126.8, 120.1, and 114.4 F g^−1^ at the current density of 1, 2, 3, 4, 5, 6, 8, and 10 A g^−1^, respectively. The change in its *C* as a function of current density is also plotted in Fig. 7e. Obviously, the *C* of the Ni_3_Si_2_O_5_(OH)_4_/RGO microsphere electrode gradually drops with increasing current density. It is inferred that both the outer and inner pores and reactive sites would contribute to the electrochemical reactions at low current densities, giving rise to high *C* values, while only the external surface of the electrode material is involved in the charge/discharge processes at high current densities, thus resulting in the diminishment of the *C* value [4, 29]. Compared with its maximum *C* at the current density of 1 A g^−1^, its *C* at 5 and 10 A g^−1^ maintains as high as 74.1 and 63.9% of the initial one, respectively, indicating prominent rate capability. However, for the bare Ni_3_Si_2_O_5_(OH)_4_ microsphere electrode, the *C* at 5 and 10 A g^−1^ decreases to only 57.2 and 47.2% of that at 1 A g^−1^, respectively, exhibiting inferior rate capability (Additional file 1: Figure S3). It is assumed that two reasons are responsible for the capacitance enhancement and rate capability improvement of the Ni_3_Si_2_O_5_(OH)_4_/RGO microsphere electrode. On the one hand, Ni_3_Si_2_O_5_(OH)_4_/RGO microspheres feature a porous hollow structure with a high-level hierarchy and larger specific surface area, which is quite favorable for the rapid transport and adsorption of electrolyte ions inside the electrode material. On the other hand, benefiting from the hybridization of Ni_3_Si_2_O_5_(OH)_4_ with RGO, the electronic conductivity is significantly improved, thus facilitating more effective electron transport within the Ni_3_Si_2_O_5_(OH)_4_/RGO microsphere electrode.

To further interpret the enhanced electrochemical behavior of the Ni_3_Si_2_O_5_(OH)_4_/RGO microsphere electrode, EIS measurements on Ni_3_Si_2_O_5_(OH)_4_/RGO and bare Ni_3_Si_2_O_5_(OH)_4_ microsphere electrodes were conducted, and the corresponding Nyquist plots are presented in Fig. 7f. Both of them show a depressed semicircle in the high-frequency region together with a straight line in the low-frequency region. In the high-frequency region, the intercept at the real axis and the diameter of the semicircle represent the equivalent series resistance (*R*_s_) of the electrode and the charge transfer resistance (*R*_ct_) at the electrode/electrolyte interface, respectively [32–34]. Apparently, compared with the bare Ni_3_Si_2_O_5_(OH)_4_ microsphere electrode, the Ni_3_Si_2_O_5_(OH)_4_/RGO microsphere electrode possesses much smaller *R*_s_ and *R*_ct_ values, which are indeed indicative of its better electronic conductivity and allow for faster electron transport within the electrode matrix [32–34]. In the low-frequency region, the straight line reflects the Warburg impedance, which can be used to describe the diffusive resistance of electrolyte ions [32–34]. The Ni_3_Si_2_O_5_(OH)_4_/RGO microsphere electrode shows a higher slope than the bare Ni_3_Si_2_O_5_(OH)_4_ microsphere electrode in the linear part, suggesting more rapid ion diffusion inside it [32–34]. These EIS findings further support and verify the abovementioned analyses on the excellent electrochemical performances of the Ni_3_Si_2_O_5_(OH)_4_/RGO microsphere electrode.

**Fig. 8:**
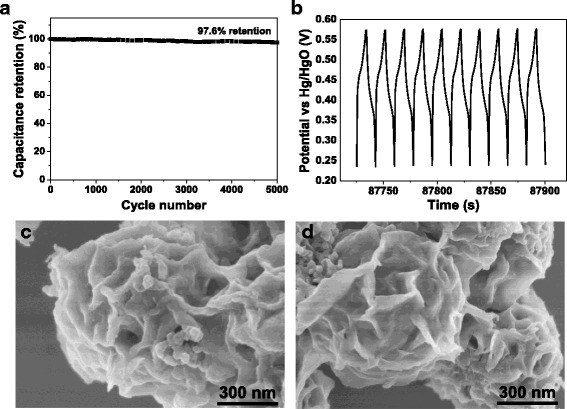
**a** Cyclic performance of Ni_3_Si_2_O_5_(OH)_4_/RGO microsphere electrode at the current density of 6 A g^–1^ for up to 5000 cycles. **b** The charge/discharge curve of Ni_3_Si_2_O_5_(OH)_4_/RGO microsphere electrode for the last 10 cycles. **c**, **d** FESEM images of Ni_3_Si_2_O_5_(OH)_4_/RGO microsphere electrode after charging/discharging for 5000 cycles

Cycle life plays a key role in the application of electrode materials in supercapacitors, since little change in their capacitance would make the supercapacitors work steadily and safely [27]. The cyclic performance of the Ni_3_Si_2_O_5_(OH)_4_/RGO microsphere electrode was determined by repetitive GCD measurements for up to 5000 cycles at the current density of 6 A g^−1^ (Fig. 8a, b). As continuous charging/discharging proceeds, the capacitance decays quite slowly (Fig. 8a), and the shape of the GCD curve for the last 10 cycles remains good enough (Fig. 8b). The capacitance retention of the electrode even reaches up to 97.6% after the whole tests, which is preferable or comparable to a number of nickel-based supercapacitor electrode materials reported previously (Table 1). Besides, the Ni_3_Si_2_O_5_(OH)_4_/RGO microsphere electrode after such tests was subjected to FESEM examinations as well, which disclosed the hierarchical porous architecture with spherical morphology freed from significant collapse and deformation (Fig. 8c, d). The structural integrity during the repetitive charging/discharging process largely contributes to the capacitance retention and convincingly demonstrates the splendid cycling stability, durability, and application potential in practical supercapacitors.

**Table 1:** Comparison of cycling stability between Ni_3_Si_2_O_5_(OH)_4_/RGO composite hollow microspheres and some of other reported nickel-based electrode materials. All these data were obtained in three-electrode systems

Electrode materials	Current density (A g^–1^)	Number of cycles	Capacitance retention (%)	Ref.
Ni_3_Si_2_O_5_(OH)_4_/RGO composite hollow microspheres	6	5000	97.6	This work
NiO nanoparticles	2	1000	60.6	[35]
NiO nanosheet hollow spheres	3	1000	91	[36]
Nanoporous Ni(OH)_2_	1	2000	92.3	[37]
Porous Ni(OH)_2_/NiOOH composite film	2	1000	81	[38]
Doughnut-like Ni(OH)_2_–Co(OH)_2_ composites	5	1000	84.7	[39]
Ni(OH)_2_–Ni nanohybrids	5	2000	98.4	[40]
Mesoporous NiCo_2_O_4_ nanosheets	2	2400	94	[41]
Urchin-like NiCo_2_O_4_ nanostructures	8	2000	90.8	[42]
Mesoporous Ni_0.3_Co_2.7_O_4_ hierarchical structures	0.625	3000	98.1	[43]
RGO/NiCo_2_O_4_ nanoflake composites	4	2000	90.8	[44]
NiCo_2_S_4_ ball-in-ball hollow spheres	5	2000	87	[45]

## Conclusions

In summary, GO-encapsulated SiO_2_ microspheres were prepared by sonication-assisted interfacial self-assembly of tiny GO sheets on positively charged SiO_2_ microspheres. By employing the resulting SiO_2_/GO composite microspheres as the template and silicon source, Ni_3_Si_2_O_5_(OH)_4_/RGO composite hollow microspheres were one-pot hydrothermally synthesized, which possessed unique hierarchical porous architecture with a large surface area. When used as a supercapacitor electrode material, Ni_3_Si_2_O_5_(OH)_4_/RGO composite hollow microspheres delivered a maximum specific capacitance of 178.9 F g^−1^ at the current density of 1 A g^−1^, which was better than that of currently developed contrastive bare Ni_3_Si_2_O_5_(OH)_4_ hollow microspheres and bare RGO material, exhibiting enhanced supercapacitive property. Of note, the Ni_3_Si_2_O_5_(OH)_4_/RGO microspheres had salient rate capability and long-term cycling stability, which maintained 97.6% of the initial capacitance after continuous charge/discharge for up to 5000 cycles, displaying a remarkably supercapacitive advantage over lots of other reported nickel-based materials. These results testify that Ni_3_Si_2_O_5_(OH)_4_/RGO composite hollow microspheres are a promising candidate for high-performance energy storage devices and systems. Moreover, we also anticipate that the present self-template synthetic strategy would be adopted to develop more and more other metal silicate-based materials with distinct morphologies and structures for important applications in various fields.

## Additional file

Additional file 1: The experimental details for synthesis of SiO_2_ microspheres and SiO_2_/GO composite microspheres. Figure S1 (a, b) FESEM images of pristine SiO_2_ microspheres at low and high magnifications, respectively. (c, d) FESEM images of SiO_2_/GO composite microspheres at low and high magnifications, respectively. Figure S2 FESEM images of Ni_3_Si_2_O_5_(OH)_4_/RGO hybrid material synthesized in the absence of PVP. Figure S3 (a) GCD curves of bare Ni_3_Si_2_O_5_(OH)_4_ hollow microspheres measured at a set of varied current densities. (b) Specific capacitance of bare Ni_3_Si_2_O_5_(OH)_4_ hollow microsphere electrode deduced from the GCD curves depicted in (a) as a function of current density. (DOCX 5399 kb)

## Abbreviations

BE: Binding energy
BET: Brunauer–Emmett–Teller
CV: Cyclic voltammetry
EDS: Energy-dispersive spectroscopy
EIS: Electrochemical impedance spectroscopy
FESEM: Field emission scanning electron microscopy
GCD: Galvanostatic charge/discharge
GO: Graphene oxide
HRTEM: High-resolution transmission electron microscopy
NMP: *N*-Methyl-2-pyrrolidone
PDDA: Poly(diallyldimethylammonium) chloride
PVDF: Polyvinylidene fluoride
PVP: Polyvinylpyrrolidone
RGO: Reduced graphene oxide
STEM: Scanning TEM
TEM: Transmission electron microscopy
XPS: X-ray photoelectron spectra
XRD: X-ray diffraction
