# Supplementary material for: Self-templated Synthesis of Nickel Silicate Hydroxide/Reduced Graphene Oxide Composite Hollow Microspheres as Highly Stable Supercapacitor Electrode Material
Source: Nanoscale Res Lett. 2017 May 4;12:325. doi: 10.1186/s11671-017-2094-9 (PMC5418184; doi:10.1186/s11671-017-2094-9)
Supplement: Additional file 1: — The experimental details for synthesis of SiO2 microspheres and SiO2/GO composite microspheres. Figure S1 (a, b) FESEM images of pristine SiO2 microspheres at low and high magnifications, respectively. (c, d) FESEM images of SiO2/GO composite microspheres at low and high magnifications, respectively. Figure S2 FESEM images of Ni3Si2O5(OH)4/RGO hybrid material synthesized in the absence of PVP. Figure S3 (a) GCD curves of bare Ni3Si2O5(OH)4 hollow microspheres measured at a set of varied current densities. (b) Specific capacitance of bare Ni3Si2O5(OH)4 hollow microsphere electrode deduced from the GCD curves depicted in (a) as a function of current density. (DOCX 5399 kb) [file 11671_2017_2094_MOESM1_ESM.docx]

**Additional File**

**Self-Templated Synthesis of Nickel Silicate Hydroxide/Reduced Graphene Oxide Composite Hollow Microspheres as Highly-Stable Supercapacitor Electrode Material**

Yanhua Zhang, Wenjie Zhou, Hong Yu, Tong Feng, Yong Pu, Hongdong Liu, Wei Xiao* and Liangliang Tian*

*Research Institute for New Materials Technology, Chongqing University of Arts and Sciences, Yongchuan Chongqing 402160, China*

***** Corresponding authors.

E-mail addresses: showame@aliyun.com (W. Xiao); tianll07@163.com (L. Tian)

**Synthesis of SiO_2_ microspheres and SiO_2_/GO composite microspheres**

SiO_2_ colloids were prepared according to a modified Stöber method. In a typical process, 100 mL of ethanolic solution containing 0.04 mol tetraethyl orthosilicate was mixed with another 100 mL of ethanolic solution containing 3.4 mol water and 0.5 mol ammonia under moderate stirring, and the reaction was allowed to proceed overnight to produce SiO_2_ colloidal microspheres, which were then harvested by centrifugation, washing and drying. Afterwards, 1 g of as-fabricated SiO_2_ microspheres were ultrasonically dispersed in 100 mL of water to form a homogeneous suspension. The resulting suspension was added into 100 mL of aqueous poly(diallyldimethylammonium) chloride (PDDA) solution (1 wt%), and the mixture was vigorously stirred overnight to yield polyeletrolyte-decorated SiO_2_ microspheres (i.e., PDDA-modified SiO_2_ microspheres with positive surface charge), which were collected by centrifugation, washing and drying. To fabricate SiO_2_/GO composite microspheres, a suspension of tiny GO sheets with the concentration of 4 mg mL^−1^ was centrifuged at a high speed of 16,000 rpm for 5 min to remove any aggregates, and the upper clear GO suspension was collected, sonicated and used for the following sonication-assisted interfacial self-assembly process. Subsequently, 100 mL of aqueous suspension of PDDA-modified SiO_2_ microspheres (4 mg mL^−1^) was dropwise added into another 100 mL of the above pre-treated aqueous GO suspension within 30 min under violent sonication (160 W). The resulting mixture was subjected to intense sonication for another 30 min to ensure the sufficient interfacial self-assembly of tiny GO sheets on the positively charged SiO_2_ microspheres through electrostatic interaction, giving rise to the GO wrapped SiO_2_ microspheres (i.e., the SiO_2_/GO composite microspheres), which were then separated from the unreacted GO sheets by centrifugation at a relatively low speed of 8,000 rpm for 5 min. At last, the collected yellow brown precipitates were dried in a vacuum oven after washing with abundant water and centrifugation.


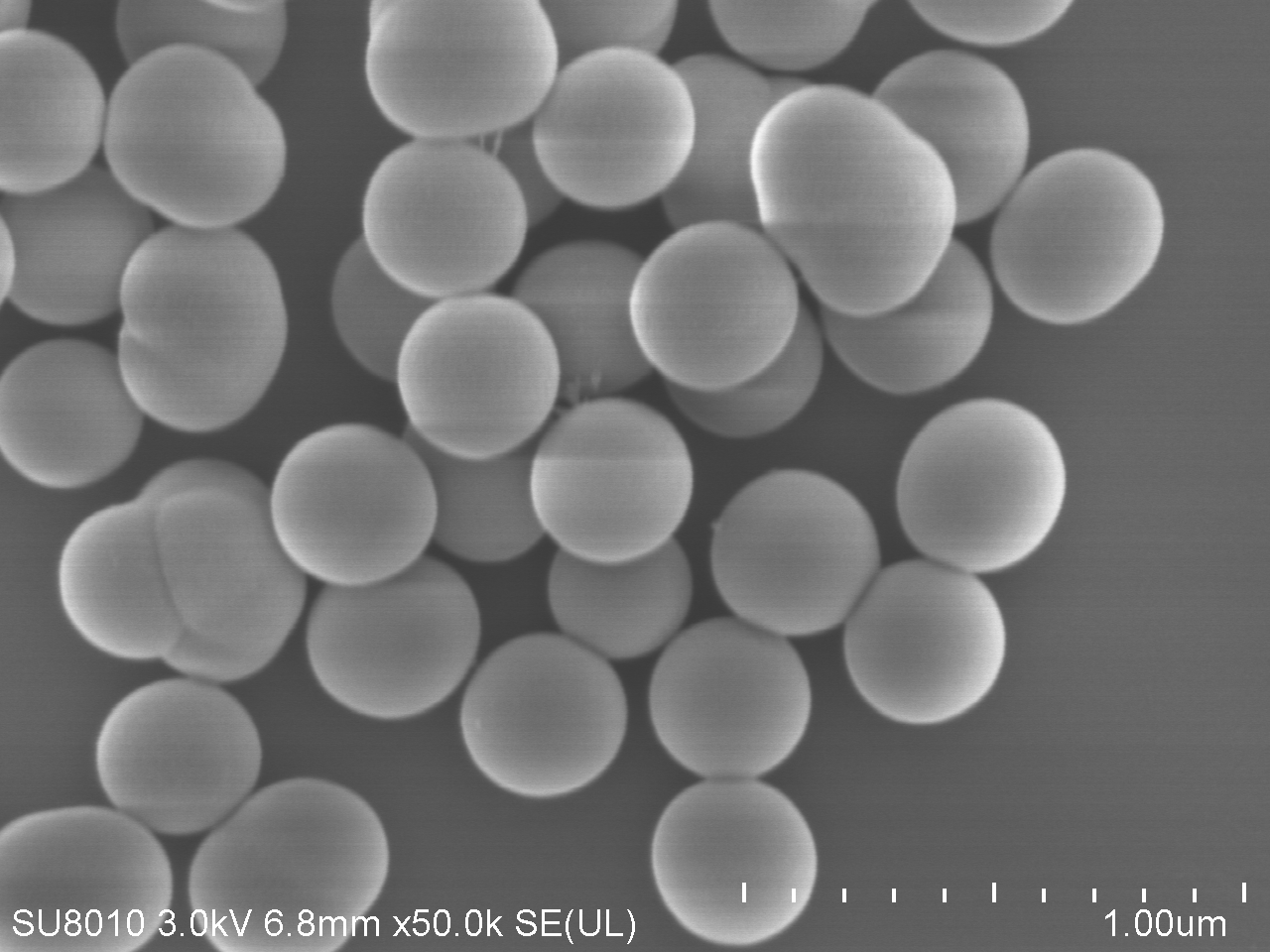

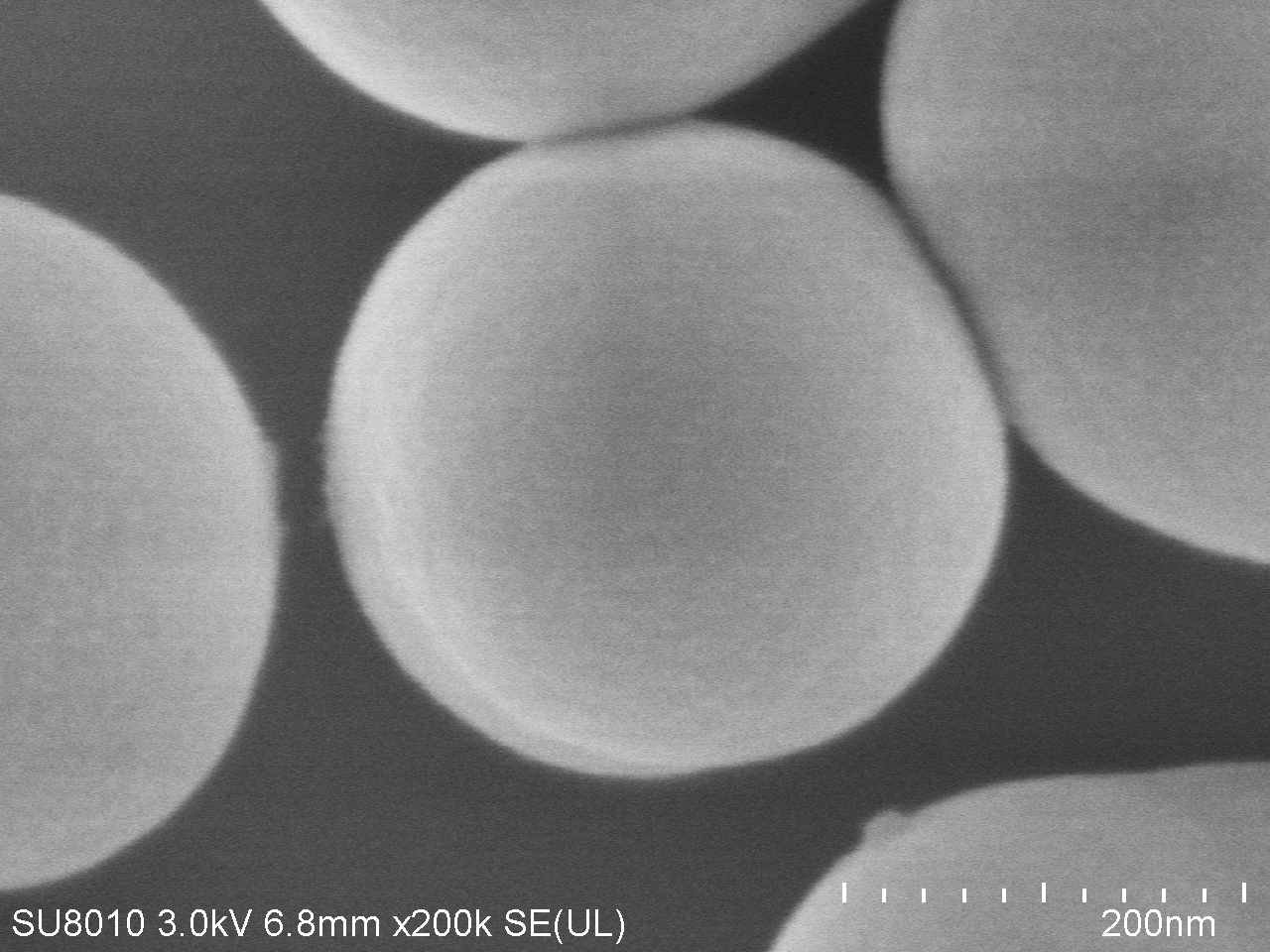

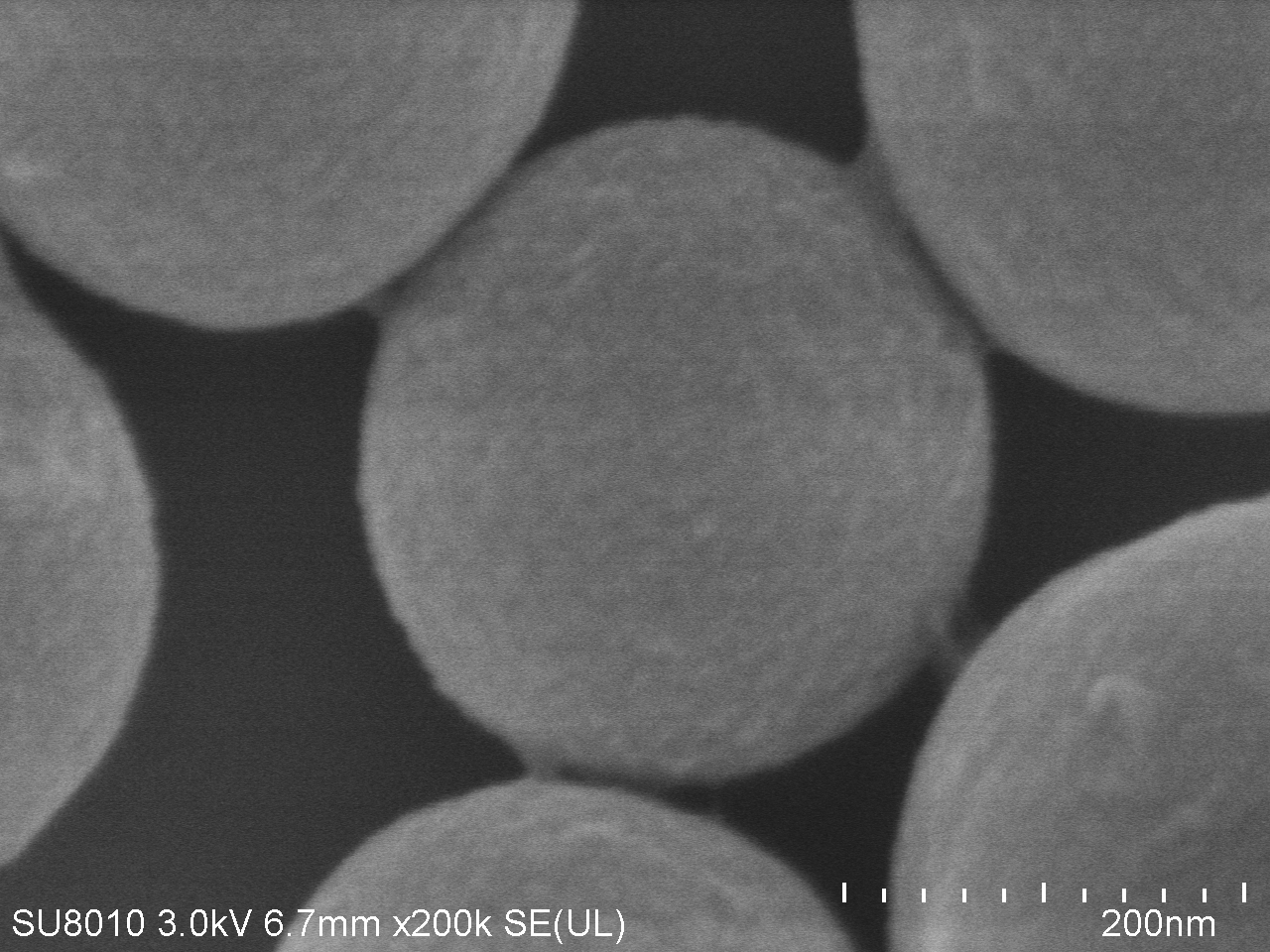

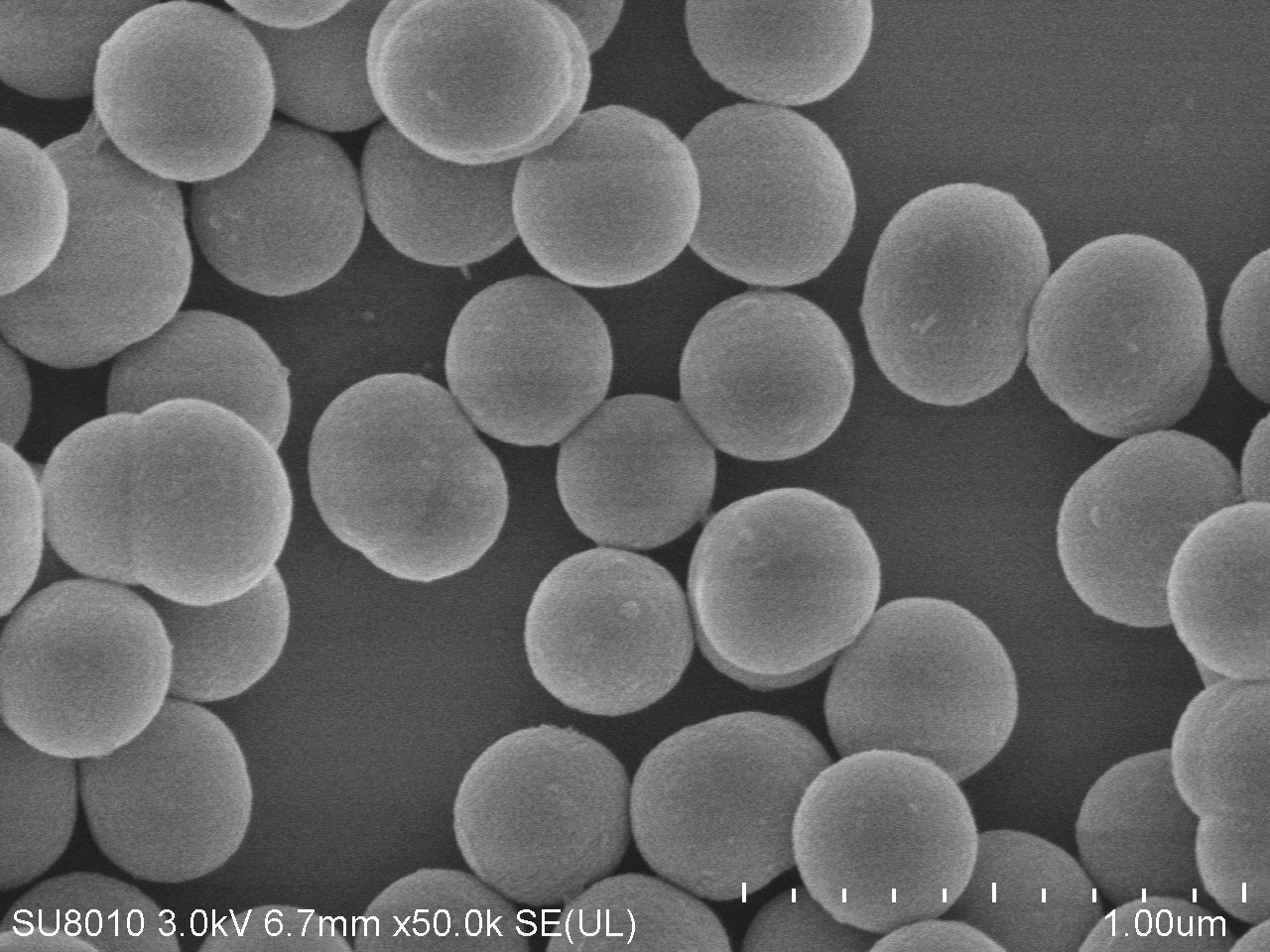


**(a)**

**(b)**

**(c)**

**(d)**

**500 nm**

**100 nm**

**500 nm**

**100 nm**

**Fig. S1** (**a**,**b**) FESEM images of pristine SiO_2_ microspheres at low and high magnifications, respectively, showing the smooth external surface. (**c**,**d**) FESEM images of SiO_2_/GO composite microspheres at low and high magnifications, respectively, exhibiting the relatively rougher outer surface.


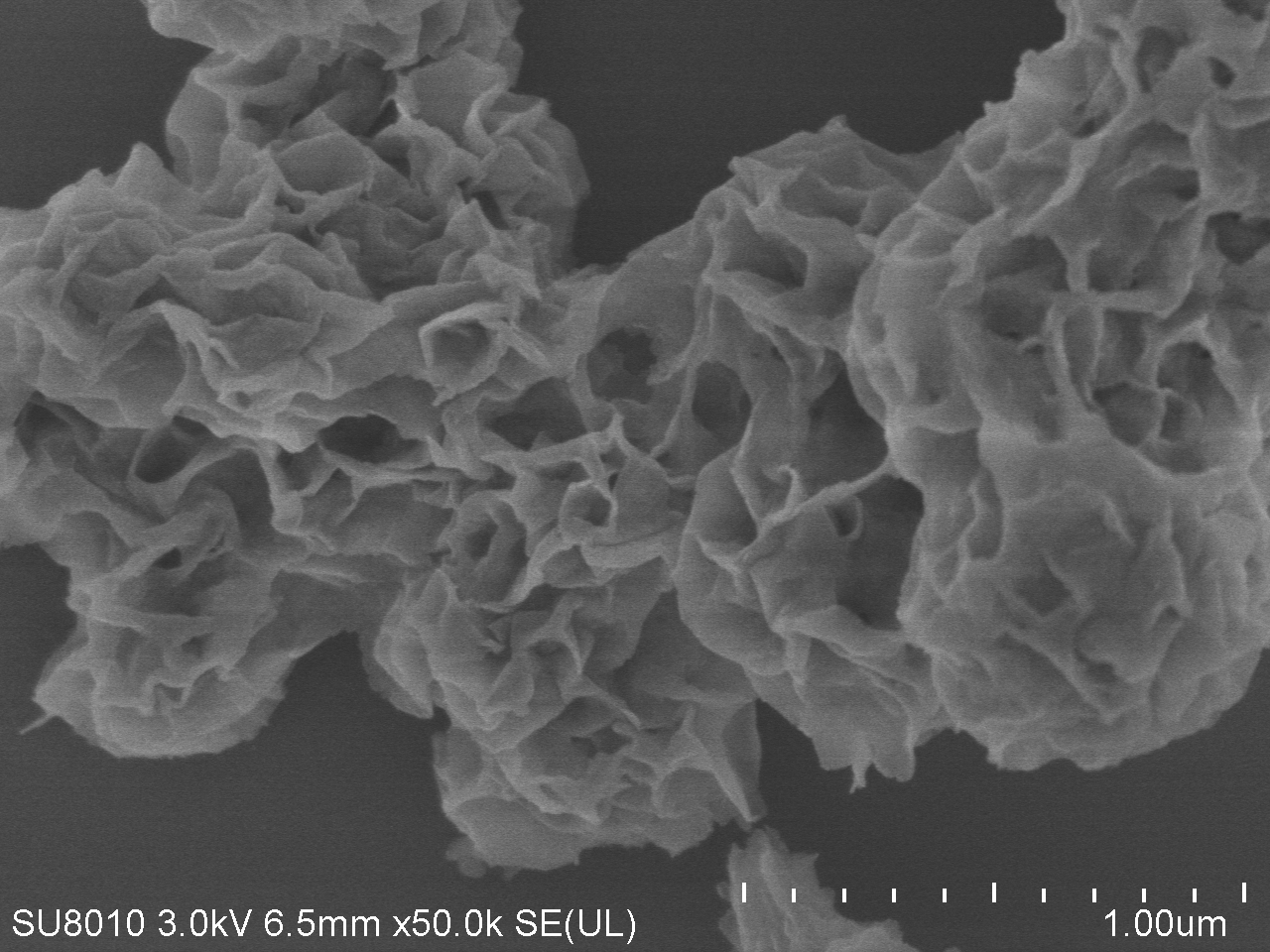

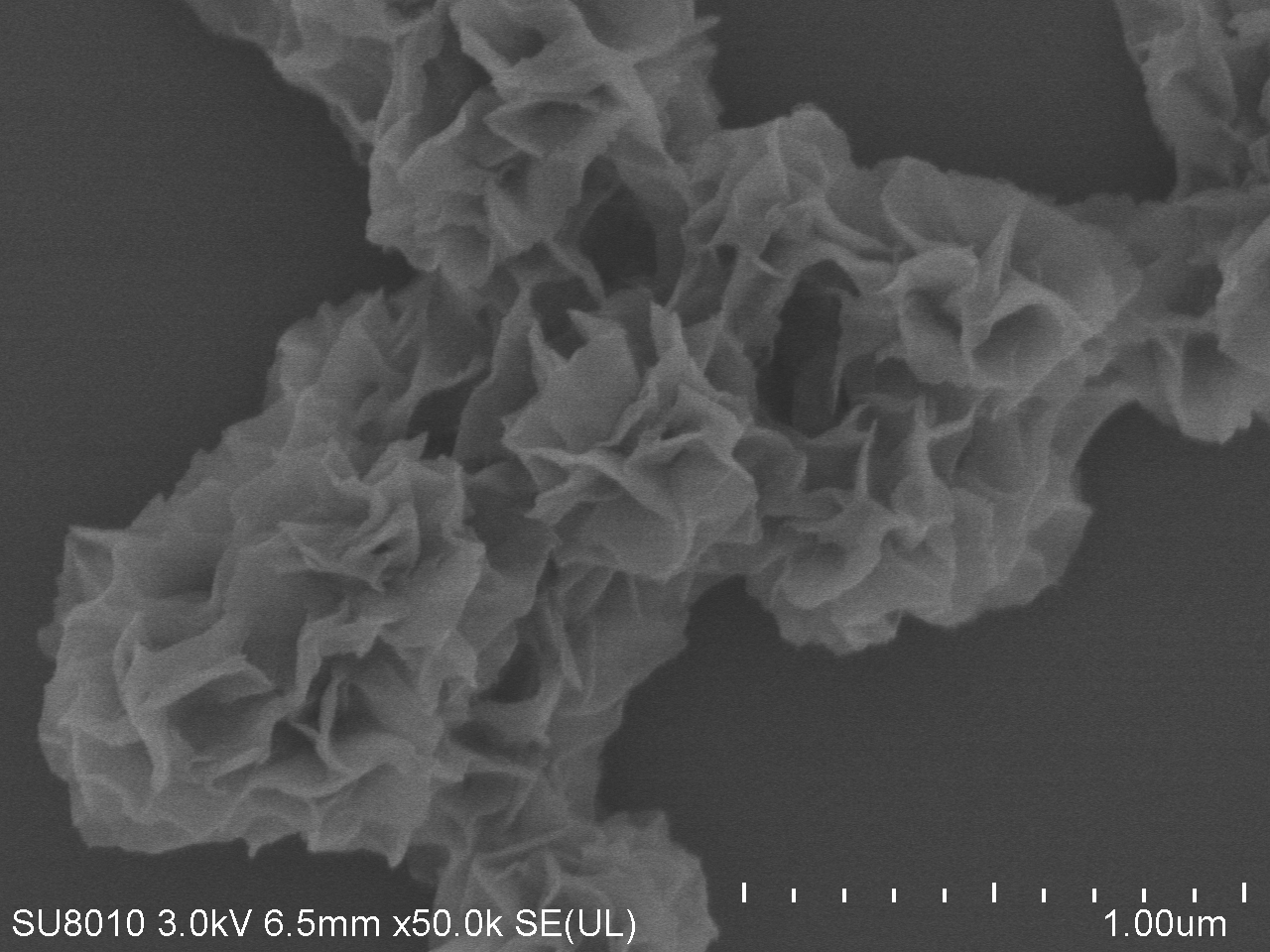


**500 nm**

**500 nm**

**Fig. S2** FESEM images of Ni_3_Si_2_O_5_(OH)_4_/RGO hybrid material synthesized in the absence of PVP.

**(b)**

**(a)**

**Fig. S3** (**a**) GCD curves of bare Ni_3_Si_2_O_5_(OH)_4_ hollow microspheres measured at a set of varied current density. (**b**) Specific capacitance of bare Ni_3_Si_2_O_5_(OH)_4_ hollow microsphere electrode deduced from the GCD curves depicted in (**a**) as a function of current density.
